# Outcomes of Idiopathic Full-Thickness Macular Hole Surgery: Comparing Two Different ILM Peeling Sizes

**DOI:** 10.1155/2020/1619450

**Published:** 2020-08-18

**Authors:** Alireza Khodabande, Alireza Mahmoudi, Hooshang Faghihi, Fatemeh Bazvand, Ebrahim Ebrahimi, Hamid Riazi-Esfahani

**Affiliations:** Retina Service, Farabi Eye Hospital, Tehran University of Medical Science, Tehran, Iran

## Abstract

**Purpose:**

This study aimed to show the impact of different extents of internal limiting membrane (ILM) peeling on visual and anatomical outcomes following idiopathic full-thickness macular hole (FTMH) surgery.

**Methods:**

In this single-center prospective study, patients with idiopathic FTMH underwent standard pars plana vitrectomy with two different extents of ILM peeling: 2-disc diameters (DD) or 4 DD. The main outcome measures were the closure rate of the holes based on optical coherence tomography (OCT) findings at three months after surgery.

**Results:**

Forty eyes from 39 patients were enrolled in the study. After three months, anatomical closure was achieved in 78% and 76% eyes in 2 DD peel and 4 DD peel groups, respectively. From 29 eyes with macular hole index (MHI) ≤ 0.5, type 1 closure was achieved in 42% eyes receiving a 2 DD ILM peel, compared to 66% eyes receiving a 4 DD peel (*p*=0.041). In comparison, this significant difference was not seen in the subgroup of MHI > 0.5 (*p*=061). In the subgroup of subjects with baseline MHI ≤ 0.5, visual improvement was significantly more in eyes with 4 DD ILM peeling (*p*=0.034), which was not seen in the MHI > 0.5 subgroup (*p*=0.61).

**Conclusion:**

In patients with idiopathic full‐thickness macular hole (MHI ≤ 0.5), a larger ILM peel of 4 DD appears to yield better anatomical outcomes than a more limited 2 DD peel.

## 1. Background

An idiopathic FTMH is a foveal defect, which is responsible for central vision loss [1]. When left untreated, it often leads to severe central vision loss to the levels of 20/200 or worse [2]. By the introduction of the modern vitrectomy, FTMH has now become a surgically treatable disease [3]. Over the past decade, several surgical technique modifications have been introduced [4].

A surgical approach for the management of idiopathic macular hole (MH) is pars plana vitrectomy (PPV) and a combination of adjuvant techniques, including internal limiting membrane (ILM) peeling, gas tamponade, and postoperative prone posturing [5].

Although there has been controversy over the role of the ILM peeling in surgical success in the past, ILM peeling has been proven to ameliorate anatomical and functional success rates, especially in holes with a diameter larger than 300 microns [5–8]. ILM peeling has been simplified by using different dyes such as brilliant blue-green (BBG) [9]. It has been shown that BBG assisted ILM peeling could contribute to better visual acuity outcomes than other dyes in patients with FTMH [8, 10].

Although ILM itself has no contractile characteristics, the myofibroblastic cells use this membrane as a scaffold to differentiate into contractile tissues [11]. ILM peeling reduces the tangential forces by these tissues on the fovea. Also, it decreases the chance of macular hole reopening by removing this scaffold for postoperative retinal surface glial cell proliferation [2, 5, 10]. Some surgeons reserve this maneuver to treat large FTMH while others use it routinely in all cases [12].

Although ILM peeling as a part of surgical treatment for FTMH has become widely accepted, there is no consensus about the optimal size of ILM peeling [13]. The impact of different extents of ILM peeling on anatomic and functional outcomes of FTMH surgery is not clear [12]. Different investigators have described different extents of the peels, from measuring only a disk diameter centered on the fovea to extended peels about four-disc diameter (about 6.5 mm) to the vascular arcades [14, 15].

Significant improvement of postmacular hole surgery metamorphopsia has been reported with a larger extent of ILM peelings. Nevertheless, some complications such as macular thinning and retinal nerve fiber layer (RNFL) injury may be more prevalent [13, 16, 17].

This study aimed to show the impact of different peeling sizes on closure rate, visual outcomes, and anatomical restoration of outer retina layers following FTMH surgery.

## 2. Methods

This is a single-center prospective study of patients who were diagnosed with idiopathic FTMH at Farabi eye hospital and were enrolled between July 2017 and October 2019. All the patients underwent standard pars plana vitrectomy (PPV) with ILM peeling to have their macular holes treated. All the patients provided written informed consent, and the study was performed with the approval of the Institutional Review Board and ethics committee of Tehran University of Medical Science and complied with the guidelines of the Declaration of Helsinki.

We excluded eyes with a traumatic FTMH, or high myopia-associated MH (defined as eyes with a myopic refractive error of greater than 6.00 diopters), a retinal detachment-associated FTMH, and long-standing macular holes (defined as a duration of 6 months or more based on previous OCT or patient's symptoms). Patients with other causes of decreased vision (e.g., uveitis, glaucoma, corneal opacity, age-related macular degeneration, and diabetic retinopathy), history of any intraocular surgery other than uncomplicated cataract surgery, and eyes with poor image quality were also excluded.

Before standard pars plana vitrectomy, eligible patients were randomly allocated 1 : 1 ratio to either group 2 DD or 4 DD:

1. 2 DD peel group included the eyes undergoing ILM peeling with a radius of one-optic-disc diameter (approx. 3.6 mm)
2. 4 DD peel group included the eyes undergoing ILM peeling with a radius of two-optic-disc diameter (approx. 7.2 mm)

Baseline demographic data including gender, age, and lens status were recorded for each subject.

All the enrolled patients underwent a complete preoperative baseline evaluation, followed up 3 months after surgery, including examination for best-corrected visual acuity (BCVA), slit-lamp examination, Goldmann applanation tonometry, dilated fundus examination, and horizontal OCT scans through the fovea with spectral-domain OCT (SD_OCT) (Spectralis HRA-OCT, Heidelberg Engineering, Heidelberg, Germany).

All clinical personnel were masked as to which patients were in the 2DD or 4DD groups.

### 2.1. FTMH Measurements

**Figure 1:**
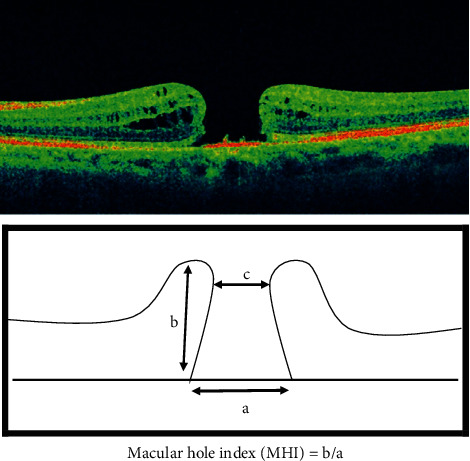
The macular hole index (MHI) [18]. Top: optical coherence tomography (OCT) cross-sectional image of a macular hole. Bottom: diagram showing the base diameter of the hole (a), hole height (b), and minimum diameter of the hole (c), as measured by OCT. The MHI is defined as b/a.

An experienced technician performed all OCT scans. The minimum diameters of FTMH (minimum linear dimension of FTMH) and MHI were measured (defined as the ratio of the hole height to the basal hole diameter: length of the retinal pigment epithelium (RPE) not in contact with the photoreceptors) (Figure 1).

According to previous studies, the holes were divided into more or less than 400 microns based on the minimum diameter of FTMH, and all the holes were divided into smaller or larger than 0.5 based on MHI [18, 19].

All the measurements were done by using the built-in caliper of Spectralis mapping software, Heidelberg Eye Explorer (version 6.0c). After the surgery, the anatomical status of the macular holes was classified into 3 categories based on SD-OCT appearance [20]:

1. Macular hole closure type 1: FTMH is closed without bare RPE.
2. Macular hole closure type 2: foveal defect persists after operation, although the hole rim is attached to the RPE.
3. Open: the foveal defect persists after operation, and the edges of the hole also remain detached from the beneath RPE.

Ellipsoid zone (EZ) status was also categorized into three groups: complete resolution, interrupted (incomplete resolution), and not improved.

Two masked vitreoretinal specialists did all the measurements, and a third masked specialist made a final decision if disagreement existed.

### 2.2. Surgical Procedure

A standard 3-port 23-gauge, sutureless, pars plana vitrectomy, and gas tamponade were performed for all patients by a single surgeon (A.K). After core vitrectomy, triamcinolone assisted posterior vitreous detachment was done. Then, patients had the BBG assisted ILM peeling with a peeling diameter of 2 DD (radios of 1 DD) or 4 DD (radios of 2DD) with ILM forceps, according to the surgeon's perception, respectively. Following the complete air-fluid exchange, tamponade was done by sulfur hexafluoride (SF6 20%) in all patients, who were instructed to face-down position for at least 3-4 days following surgery.

The patients with significant cataract enough to preclude the ILM peeling would receive combined phacoemulsification with intraocular lens implantation and PPV. For patients with persisting macular hole after PPV, they were advised to undergo a second surgery with a more extended ILM peeling. Nd : YAG laser treatment was done for the eyes developing visually significant posterior capsular opacification.

The primary outcome was the proportion of eyes with complete closure of the holes based on OCT findings within each group at three months after operation. The secondary outcome measure consisted of the BCVA and anatomical outcomes difference between two groups, along with the difference between the BCVA and anatomical outcomes in subgroups when subjects were stratified by baseline macular hole minimum diameter and MHI.

### 2.3. Statistical Analysis

Continuous variables were expressed as mean (±standard deviation), and categorical variables were expressed as percentages.

Chi-square test/Fisher's exact test was used to assess the association between categorical variables. Independent *t*-test/Mann–Whitney *U* test was used to discover the significant difference of continuous variables between the two study groups.

A Chi-square test was applied to compare the structural outcomes between the groups based on OCT findings. The independent sampled *t*-test was used to analyze the postoperative visual outcomes between the groups. Fisher's exact tests were also utilized in subgroup analysis divided by the cut-off value of MHI or macular hole diameter to compare the anatomical outcomes between the two groups. Logistic regression was used for multivariable analysis.

All analyses were conducted using SPSS software version 22.0 (SPSS Inc., Chicago, IL, USA). A *P* value of less than 0.05 was considered statistically significant.

## 3. Results

Forty-four eyes from 43 patients were enrolled in this study. From the 44 eyes, four eyes from 4 patients were excluded due to low image quality or failure to follow-up before finishing the three months after surgery. Forty eyes of 39 patients were randomized into either the 2 DD (*n* = 19, 47%) or 4 DD (*n* = 21, 52%) ILM peeling group.

The mean preoperative minimum macular hole diameters in 2 DD peel group and 4 DD peel group were 466.97 ± 161.88 *μ*m and 522.90 ± 126.61 *μ*m, respectively (*p*=0.23). The mean base diameters in 2 DD peel group and 4 DD peel group were 1048.11 ± 220.58 *μ*m and 975.33 ± 254.56 *μ*m, respectively (*p*=0.28). The macular hole index (MHI) was not significantly different between the groups (*p*=0.43). The mean BCVA in groups 2 DD and 4 DD were 0.97 ± 0.37 and 0.99 ± 0.20, respectively, based on log MAR (*p*=0.12) (Table 1).

Among the eyes undergoing surgery, 30 eyes were phakic (75%) and 10 were pseudophakic (25%). There was no significant difference in the number of pseudophakic or phakic eyes in each group. Cataract surgery with intraocular lens implantation was done just before vitrectomy in one session in all phakic eyes. Combination surgery did not reveal a significant influence on macular hole closure (*p*=0.47). Posterior vitreous detachment (PVD) was present in 10 eyes (25%) before surgery, while PVD was induced during surgery in the remaining eyes.

Anatomical closure was achieved in 78% (*n* = 15/19) and 76% (*n* = 16/21) eyes in 2 DD peel group and 4 DD peel group, respectively (−2.3% difference, 95% confidence interval (CI) : −9.2%–4.6%; *p*=0.83). The closure type 1 was 52% (*n* = 10/19) in 2 DD peel group versus 76% (*n* = 16/21) in 4 DD peel group. Type 1 closure was achieved significantly more in 4 DD peel group (23.6% difference; 95% CI : 13.9%–33.3%; *p*=0.041). Overall, MH in 9 cases were not closed (anatomic failure) after the first surgery, and a second surgery was recommended for them.

Logistic regression analysis revealed that the ILM peeling size independently from other covariate had a significant effect (odds ratio (OR) = 2.74; 95% CI : 1.07–6.99; *p*=0.035) on the type 1 MH closure rate.

In macular holes with minimum linear dimension equal to or greater than 400 microns, closure type 1 occurred in 6/13 (46%) of the eyes with 2 DD peeling size, in contrast to 13/17 (76%) eyes with 4 DD peeling size. This difference was statistically significant (30.3% difference; 95% CI : 21.9%–38.7%; *p*=0.031). In macular holes with a minimum linear dimension less than 400 microns, all holes were closed successfully after surgery in both groups, and the rate of closure type 1 was not different significantly between these two groups 4/6 in 2 DD peel group versus 3/4 in 4 DD peel group (8.4% difference; 95% CI : 4.2%–12.6%; *p*=0.42) (Table 2).

Based on the previous studies, an MHI cut-off value of 0.5 was chosen to evaluate the closure rate for clinical application in each peeling group. The comparison analysis between the two groups was done on the subgroups of subjects with MHI values below and above the 0.5 cut point.

After three months, based on Fisher's exact test, from 29 eyes with MHI ≤ 0.5, type 1 closure was achieved in 6/14 eyes (42%) receiving a 2 DD ILM peel, compared to 10/15 eyes (66%) receiving a 4 DD peel (23.8% difference; 95% CI :17.3%–30.3%; *p*=0.041). In contrast, in the subgroup of patients with MHI more than 0.5, type 1 closure was achieved in 4/5 eyes (80%) in the 2 DD group versus 6/6 (100%) in the 4 DD group (*p*=0.61) (Table 3).

The status of twenty-one eyes showed an interrupted EZ 3 months after the surgery while 13 eyes revealed no EZ after 3 months in the central fovea. From 6 eyes with complete postoperative EZ, 5/19 (26%) eyes were in 2 DD peel group, and 1/21 (4%) eye was in 4 DD peel group (*p*=0.028). Nevertheless, the eyes with no central EZ were significantly more in 2 DD peel group [8/19 (42%) eyes in 2 DD peel group vs. 5/21 (23%) eyes in 4 DD peel group (*p*=0.032)].

Preoperative mean logMAR BCVA was 0.98 ± 0.35, which was significantly improved to 0.61 ± 0.46 logMAR 3 months after surgery (*p*=0.0012). The mean BCVA improvement showed no difference in month 3 between the two groups (0.361 logMAR vs. 0.381 logMAR, 0.02 difference; 95% CI : 0.007–0.033; *p*=0.904). BCVA improvement was significantly higher in FTMH with a minimum diameter of fewer than 400 microns than larger holes (0.54 logMAR vs. 0.30 log MAR, 0.24 difference; 95% CI : 0.13–0.35; *p*=0.02) (Table 4).

Although BCVA improvement was higher across the 4 DD peel group (0.36 logMAR) than in 2 DD peel group (0.25 logMAR) among eyes with a minimum diameter equal to or greater than 400 microns, this difference was not significant (0.11 difference; 95% CI, 0.03–0.19; *p*=0.59).

BCVA improvement was significantly correlated with macular hole postoperative anatomical status as with closure type 1 (*p*=0.0091) and EZ status (*p*=0.021). Eyes with no improvement in EZ status had significantly worse BCVA than eyes with complete or interrupted EZ (*p*=0.023 and 0.012).

Three months after surgery, in the subgroup of subjects with baseline MHI ≤ 0.5, there was a significant difference between the two ILM peeling groups based on the BCVA : 2 DD group (0.82 ± 0.21), 4 DD group (0.53 ± 0.27) (−0.29 difference; 95% CI : −0.41–−0.17; *p*=0.034) (*p*=0.034) although, in the MHI > 0.5 subgroup, BCVA revealed no significant difference between the two ILM peeling groups (−0.11 difference; 95% CI : −0.21–0.01; (*p*=0.61) (Table 5).

## 4. Discussion

Based on this interventional case series on patients with FTMH, although macular hole closure (type 1 + type 2) was not significantly different between 2 DD and 4 DD peeling groups, type 1 closure was found significantly more in 4 DD peel group. In macular holes with a minimum linear dimension equal to or greater than 400 microns or with MHI ≤ 0.5, type 1 macular hole closure occurred significantly more in the 4 DD peeling group. This difference was not observed in the eyes with macular holes with a minimum linear dimension smaller than 400 or MHI greater than 0.5.

Glial and myofibroblastic cells use ILM as a scaffold for proliferation and differentiation into contractile tissues, which is thought to be essential in the pathogenesis of MH formation [21]. Therefore, ILM peeling is responsible for neutralizing these tangential traction forces and for increasing retinal compliance, allowing the retina to move more freely to assist MH closure [6]. Lois et al [14]. found that extended and more complete ILM peeling could enhance the chance of MH closure. It has been shown that extended ILM peeling in a second surgery for patients with unsuccessful previous macular hole surgery leads to success in FTMH closure and visual acuity improvement [22, 23]. Yek et al. concluded that visual acuity gain after two years was significantly higher in eyes undergoing secondary surgery with extended ILM peeling after the first surgery failure than the eyes that were followed without any secondary intervention [23].

Anatomical closure was achieved in 78.5% and 76.2% eyes in 2 DD peel group and 4 DD peel group, respectively. Our success rate was lower than the previous studies; it maybe due to our mean macular hole diameter that was very large or smaller sample size compared to other studies. Indeed, we have studied on large macular holes. The mean preoperative minimum macular hole diameters in 2 DD peel group and 4 DD peel group were 466.97 ± 161.88 *μ*m and 522.90 ± 126.61 *μ*m, respectively. The mean preoperative minimum macular hole diameter in both groups was 494.93 ± 143.28.

We observed that type 1 closure as a closing without foveal defect of the neurosensory retina was achieved significantly more in the extended peel group. It is presumed that the residual ILM with a membrane on its surface may act as a traction force that pulls the retina toward itself. Therefore, in cases with 4 DD ILM peeling, the ridge of the remnant ILM is more distant from the margins of the macular hole, and these tractional forces have less effect on the macular hole status, thereby improving reconstruction. This phenomenon may be more prominent in larger macular holes with lower MHI [16, 24, 25].

However, regarding the role of ILM in retinal function as a footplate of Müller cells and the mechanical damage to retina layers during the procedure of ILM peeling itself, some concerns have been raised about potential adverse effects, particularly in case of extended peeling due to degenerative thinning of the bare retina over time [26]. Some studies found that the retina became thinner after vitrectomy with extended ILM peeling for large MH, which might be associated with the migration of paramacular tissue [16, 27].

Inner retinal defects frequently occurred once the ILM was peeled, and it was composed of dark spots in the same orientation as the optic nerve fibers [28]. Nerve fiber layer disruption was also reported after ILM peeling based on OCT findings [29]. Furthermore, the dysfunction of Müller cells has been documented by delay in the recovery of the focal macular electroretinograms b-wave after removing the ILM in the macular area [30]. The retinal sensitivity may be reduced after extended ILM peeling, notably increasing the incidence of microscotomas [31]. Larger ILM peeling may be accompanied by more interventions to pinch and grasp the ILM; it could be confirmed with more pit like inner retinal defects coursing along the nerve fiber layer using SD-OCT. Furthermore, these dimples may be enlarged in the postoperative period [32].

In this study, the postoperative BCVA improved significantly at each visit, 3 months after surgery. BCVA improvement was significantly correlated with macular hole postoperative anatomical status, such as closure type 1 and EZ status.

Steel et al [33]. observed that the larger ILM peel size associated with the shortening of the distance between fovea and disc, shortening of the macular area, and the optic nerve fiber layer dissociation. This may lead to lower postoperative visual acuity than the surgeon's expectation. Considering these observations, the authors suggested limiting the ILM peeling size.

We also observed that the complete EZ line restoration was significantly higher in the 2 DD peel group than in the 4 DD group, which may be due to less anatomical changes and macular thinning. Although the visual improvement was not different significantly between the groups as the presence of EZ was higher in 4 DD peel group, the complete EZ line was significantly higher in the 2 DD peel group.

The present study observed that in the holes with MHI less than 0.5, visual improvement was significantly higher in the 4 DD peel group, which was accompanied by more type 1 closures. It seemed that the final visual acuity in the holes with MHI < 0.5 was correlated with the proper anatomical closure type, which might be achieved with extended peeling size.

This would appear to highlight that baseline MHI is a more accurate index for the decision on ILM peeling size than MH size itself. Kusuhara et al. [18] suggested the MHI as a more powerful predictor for visual outcome following MH surgery than the macular hole diameter. In several studies, a positive association was found between MHI and postoperative visual acuity [19, 34–36].

Thus, achieving type 1 closure of MH should be a prerequisite when considering the extent of the ILM peeling. In this regard, the present study showed that the 4 DD group achieved more encouraging structural results in a subgroup of patients with MHI less than 0.5.

In a published article, multiple regression analyses showed a correlation between structural outcomes and the diameter of the MH as well as the extent of the peeling area [37]. However, this study was retrospective with a small sample. In another study, Modi et al. [38] demonstrated that there was no relationship between the ILM peeling size and type 1 closure of MHs, regardless of its size, staging, or duration. Although Modi's study was a prospective trial, some factors may be responsible for these differences between their project and the current study. First, the difference in the extent of the ILM peeling (3 mm diameter and 5 mm diameter ILM peels) may have been too small to yield a difference in closure rates. Secondly, the subgroup analysis was based on MH size and MH stage rather than MHI. As we showed earlier, MHI was a more powerful predictor for macular hole closure.

Bae et al. [16] indicated that enlarging the size of ILM peeling is beneficial to improve postoperative metamorphopsia. Based on a prospective study by Yao et al, in comparison with 2 DD ILM peeling, 4 DD ILM peeling could lead to better structural outcomes in eyes with macular hole closure index(MHCI) or hole form factor [13]. In contrast to our study, they used HFF instead of MHI. The HFF is calculated by the summation of the EZ lengths in each side of the macular hole divided by the basal hole diameter. The HFF is suggested to be positively correlated with the postoperative visual acuity. Still, this correlation is weaker than that for the basal hole diameter and minimum diameter of the macular hole [39, 40]. In this study, in contrast to our study, macular holes with a history of more than one year differed between the two groups, which might affect the study results. Another advantage of the current study in contrast to their study was that all the operations were done by a single surgeon.

There are several limitations to the current study. First, the analysis was based on three months of follow-up data, and thus our results may not reflect the long-term MH surgery results. Secondly, the present study was a single-center study with low sample size. Of the above limitations, the relatively small sample size is the most critical shortcoming of this study. Thus, a multicenter trial with a larger sample size should be considered to reevaluate our results and to reach a more exhaustive conclusion. Finally, MHI had to be manually calculated by one trained grader, and there was no automated software for calculation.

## 5. Conclusion

In conclusion, our results suggested that 4 DD ILM peeling for MHs with MHI ≤ 0.5 can reach better structural and visual outcomes in macular hole surgery. On the other hand, for the MHs with MHI > 0.5, limited ILM peeling may be adequate to achieve satisfactory anatomical and functional outcomes. Therefore, careful, individualized assessment of preoperative MH anatomy based on OCT is necessary to optimize surgical arrangements.

## Figures and Tables

**Table 1:** The baseline characteristics of the two groups.

	Group A	Group B
	(2 DD peel group)	(4 DD peel group)
Number	19	21
Male: female	10 : 09	12 : 09
Mean age	64.6 ± 6.7	67 ± 8.4
Mean minimum diameter	466.97 ± 161.88 *μ*m	522.90 ± 126.61 *μ*m
Mean base diameter	1048.11 ± 220.58 *μ*m	975.33 ± 254.56 *μ*m
Mean macular hole index (MHI)	0.36	0.4
Mean basal visual acuity	0.97 ± 0.37	0.99 ± 0.20

**Table 2:** Anatomical outcome different between groups after 3 months following stratification by macular hole diameter.

	MH ≥ 400			MH < 400		
	2 DD (*n* = 13)	4 DD (*n* = 17)	*p* value^*∗*^	2 DD (*n* = 6)	4 DD (*n* = 4)	*p* value^*∗*^
Type 1 closure	6 (46%)	13 (76%)	0.031	4	3	0.42
Type 2 closure	3 (23%)	0	N/A	2	0	N/A
Open	4 (30%)	4 (23%)	0.89	0	1 (25%)	N/A

^*∗*^Fisher exact test.

**Table 3:** Anatomical outcome different between groups after 3 months following stratification by macular hole index.

	MHI ≤ 0.5			MHI > 0.5		
	2 DD (*n* = 14)	4 DD (*n* = 15)	*p* value^*∗*^	2 DD (*n* = 5)	4 DD (*n* = 6)	*p* value^*∗*^
Type 1 closure	6 (42%)	10 (66%)	0.041	4	6	0.61
Type 2 closure	6 (42%)	0	N/A	1	0	N/A
Open	4 (28%)	5 (33%)	0.82	0	0	N/A

^*∗*^Fisher exact test.

**Table 4:** Functional outcome in the two groups after 3 months following stratification by macular hole diameter.

	Total	MH ≥ 400			MH < 400		
		2DD (*N* = 13)	4DD (*n* = 17)	*p* value^*∗*^	2DD (*n* = 6)	4DD (*n* = 4)	*p* value^*∗*^
Baseline	0.98 ± 0.35	1.01 ± 0.26	1.02 ± 0.31		0.93 ± 0.17	0.97 ± 0.21	
3 Months after surgery	0.61 ± 0.46	0.76 ± 0.31	0.65 ± 0.21	0.59	0.39 ± 0.28	0.42 ± 0.17	0.89

^*∗*^independent sampled *t*-test: visual acuity improvement after 3 months.

**Table 5:** Functional outcome in the two groups after 3 months following stratification by macular hole index.

	Total	MHI ≤ 0.5			MHI > 0.5		
		2 DD (*N* = 14)	4 DD (*N* = 15)	*p* value^*∗*^	2 DD (*n* = 5)	4 DD (*N* = 6)	*p* value^*∗*^
Baseline	0.98 ± 0.35	1.07 ± 0.32	1.04 ± 0.31		0.901 ± 0.2 7	0.96 ± 0.30	
3 months after surgery	0.61 ± 0.46	0.82 ± 0.21	0.53 ± 0.27	0.034	0.351 ± 0.41	0.43 ± 0.21	0.61

^*∗*^Independent sampled *t*-test: visual acuity improvement after 3 months.

## Data Availability

The derived data supporting the findings of this study are available from the corresponding author on request.
